# Neuroimaging in Pediatric Epilepsy

**DOI:** 10.3390/brainsci9080190

**Published:** 2019-08-07

**Authors:** Zakir Shaikh, Alcy Torres, Masanori Takeoka

**Affiliations:** 1 Department of Pediatrics, Division of Pediatric Neurology, Boston Medical Center, Boston University School of Medicine, Boston, MA 02118, USA; 2 Department of Pediatric Neurology, Children’s Hospital Boston, Harvard Medical School, Boston, MA 02115, USA

**Keywords:** pediatric epilepsy, neuroimaging, refractory epilepsy, temporal lobe epilepsy, extra-temporal lobe epilepsy

## Abstract

Pediatric epilepsy presents with various diagnostic challenges. Recent advances in neuroimaging play an important role in the diagnosis, management and in guiding the treatment of pediatric epilepsy. Structural neuroimaging techniques such as CT and MRI can identify underlying structural abnormalities associated with epileptic focus. Functional neuroimaging provides further information and may show abnormalities even in cases where MRI was normal, thus further helping in the localization of the epileptogenic foci and guiding the possible surgical management of intractable/refractory epilepsy when indicated. A multi-modal imaging approach helps in the diagnosis of refractory epilepsy. In this review, we will discuss various imaging techniques, as well as aspects of structural and functional neuroimaging and their application in the management of pediatric epilepsy.

## 1. Introduction

Epilepsy is a disease characterized by an enduring predisposition to generate epileptic seizures and by the neurobiological, cognitive, psychological, and social consequences of this condition. An epileptic seizure is considered to be associated with abnormal neuronal activity due to excessive or unsynchronized electrical discharge resulting from abnormalities in the inhibitory and/or excitatory pathway of the central nervous system. Epilepsy is a chronic condition of recurrent, unprovoked seizures which affects 1% of the world population with an annual incidence of 68/100,000 per year. In 2015, 1.2% of the US population had active epilepsy (95% CI: 1.1–1.4). This amounts to about 3.4 million people with epilepsy nationwide: 3 million adults and 470,000 children [1]. As proposed by the International League Against Epilepsy in 2010, refractory epilepsy or drug-resistant epilepsy may be defined as “failure of adequate trials of two tolerated and appropriately chosen and used antiepileptic drug schedules (whether as monotherapies or in combination) to achieve sustained seizure freedom” [2]. This kind of epilepsy is characterized by an underlying subtle structural defect, neuro-cutaneous malformations, calcified/hemorrhagic lesions and metabolic abnormalities which can be conclusively identified with the help of neuroimaging. Recent advances in neuroimaging have made it possible to localize epileptogenic foci even in refractory epilepsy through a multi-modal approach. There are many recommendations for the universal adoption of neuroimaging guidelines for the management of epilepsy, Coryell et al., in a large study, demonstrated a high yield of neuroimaging in pediatric patients with early-life epilepsy (ELE) [3].

In this article, along with conventional imaging techniques such as computed tomography (CT scan) and magnetic resonance imaging (MRI), we will plan to discuss multimodal approaches including functional neuroimaging such as positron emission tomography (PET), single photon emission computerized tomography (SPECT), magnetic resonance spectroscopy (MRS), magnetoencephalography (MEG), functional MRI (fMRI) and their application in the pediatric population. When determining the appropriate neuroimaging studies, a relevant history, physical examination, laboratory workups and electroencephalographs (EEGs) will be supportive in identifying patients in whom neuroimaging may be helpful and which studies will be useful for their management. The role of functional neuroimaging becomes particularly important in cases where an epileptogenic lesion is in close proximity to the eloquent cortex. Neuroimaging may be less useful in certain generalized epilepsy syndromes such as juvenile absence epilepsy, juvenile myoclonic epilepsy or focal epilepsy syndromes such as benign rolandic epilepsy, benign occipital epilepsy and Panayiotopoulos syndrome [4].

## 2. Imaging Modalities

Imaging techniques can broadly be classified into structural and functional neuroimaging studies. Structural imaging is essential in identifying anatomical abnormalities associated with seizure focus. Functional imaging can provide complementary information when an epileptogenic substrate is not anatomically identified or in the presence of non-concordant clinical and structural findings. Structural neuroimaging helps to identify the underlying etiology of seizures; it is of utmost importance in localization-related epilepsy. Structural neuroimaging mainly revolves around MRI and CT scans, while MRI is the obvious imaging technique of choice; CT scan has its own role in identifying calcified lesions and hemorrhage. Children with epilepsy should undergo structural neuroimaging in the presence of one or more of the features mentioned in Table 1 [5].

In particular, there are specific types of structural brain lesion that are prone to be associated with intractable epilepsy including a malformation of cortical development (including disorders of cerebral proliferation and migration such as lissencephaly, hemimegalencephaly and focal cortical dysplasia, and developmental tumors such as gangliogliomas and dysembryoplastic neuroepithelial tumors (DNET)). Also, neurocutaneous disorders such as tuberous sclerosis complex, Sturge–Weber syndrome, neurofibromatosis, and epidermal nevus syndromes could be associated with intractable epilepsy.

In addition to localizing cerebral dysfunction, functional neuroimaging also plays a vital role in the pre-surgical evaluation of patients with refractory epilepsy. It identifies abnormalities primarily through disturbances in metabolism and cerebral vascular flow. Various techniques used in functional neuroimaging are fMRI (functional MRI), single photon emission computerized tomography (SPECT), positron emission tomography (PET) and magnetic resonance spectroscopy (MRS). A combination of these imaging techniques effectively localizes the underlying lesion even in intractable epilepsy and often is helpful in surgical planning.

## 3. Computed Tomography

Computed tomography (CT scan) utilizes ionizing radiation, which generates a tremendous hard tissue contrast and an average soft tissue resolution with poor quality in temporal fossa. Although MRI has mostly restricted the use of CT scans, CT scans are performed on patients if MRI is not available; CT may not identify some symptomatic causes for epilepsy such as mesial temporal sclerosis (MTS), small tumors and small or subtle focal cortical dysplasia. The advantages of CT scans are their lower cost, ready accessibility, ease of use and excellent scanning speed, which decrease the need for sedation in pediatric patients and allows the evaluation of younger, less co-operative patients. Fast scanning speeds also make CT scan imaging helpful in an emergency and perioperative conditions. Acute imaging with CT in emergency patients with seizures can alter management, especially in patients with new onset focal neurological abnormality or persistent changes in mental status [6]. The CT scan effectively identifies calcified lesions (congenital infections), bleed, hydrocephalus, neuro-cutaneous malformations and significant structural abnormality. The CT scan is preferred over MRI in calcified brain lesions (congenital infections—cytomegalovirus infection, toxoplasmosis) and neuro-cutaneous malformations (Sturge–Weber syndrome, tuberous sclerosis).

However, a CT scan exposes patients to the risk of radiation; children are especially susceptible to radiation exposure. The radiation dose is particularly important in pediatric patients or small adults because of the increased lifetime cancer risk associated with the amount of ionizing radiation dose received per square meter of body surface. Hence, an optimal radiation dosing is followed, which should be as low as possible [6,7]. Besides this, a CT scan generates a low-resolution image which fails to detect abnormalities in up to 50% of patients with epileptogenic structural lesions. It particularly may fail to identify lesions in temporal lobe epilepsy (e.g., mesial temporal sclerosis), or small/subtle malformations of cortical development and intractable epilepsy. The overall sensitivity of CT scans is low (30%) owing to their poor resolution in temporal fossa [6,8,9].

## 4. Magnetic Resonant Imaging

MRI uses magnetic fields to generate images of the body and is non-ionizing. MRI is the imaging modality of choice, because of its superior anatomic resolution, and provides much better detail compared to the CT scan, hence identifying more abnormalities than CT [10,11]. There are few circumstances where CT may be used for its limited advantages over MRI, such as availability and for emergent care—also for assessing calcifications—but in general, MRI is preferred for evaluation in pediatric epilepsy for the reasons listed below.

MRI provides a better resolution of temporal fossa compared to the CT scan and effectively identifies lesion of the temporal region. MRI also identifies focal cortical dysplasia, mesial temporal sclerosis, small tumors, subtle changes and vascular malformations which may be missed in a CT scan [2,12]. Disadvantages of MRI are its limited accessibility (in less developed countries), slow scanning speed, high cost, motion artifacts and requirement of sedation when cooperation is limited; the advantages and disadvantages of MRI vs. CT scans are mentioned in Table 2. 

There is no universal epilepsy protocol for MRI. However, most recommendations agree on the following protocols for epilepsy (Table 3) [8,13,14]:

Imaging sequences should have thickness of around 3–4 mm with thin-slice images (2 mm) T2-weighted for subtle focal cortical dysplasia and with a 1–1.5 mm thickness for 3D T1 sequences. In the case of focal epilepsy, thinner slices may be required to identify subtle cortical malformations. The above imaging sequences should be obtained in two planes (axial and coronal) with the oblique coronal sequence for the maximal visualization of the hippocampus. Routine gadolinium contrast is not indicated; this is reserved for suspected tumors, vascular malformations, inflammation, and infections based on clinical information and non-contrast studies [5,15].

For children younger than two years of age, special sequences are required because of rapid developmental changes which may conceal the epileptogenic defect. The two most critical developmental changes occurring within the first few years of life are a reduction in total volume of water and the maturation of myelination, which allow the easy identification of the gray and white matter. Hence, with changing myelination patterns, the MRI protocol changes and should include T1 weighted images and high-resolution T2 weighted images in sagittal axial and coronal planes along with 3D sequences. Some authors also suggest the use of dual echo-Short Tau Inversion Recovery (echo-STIR) sequences instead of the T2 weighted sequence in the under two-year age group. Patients with persistent seizures and normal MRI in the first two years of life need repeat MRIs at the interval of 6 months and certainly after the more mature pattern of myelination (30 months of age) which may reveal unsuspected cortical abnormalities [5].

The advanced MRI techniques using higher magnetic field strength 3 Tesla (3 T) or 7 Tesla (7 T) and multichannel coils have significantly improved sensitivity. The use of a higher magnetic field strength and multichannel coils provides a higher signal-to-noise ratio, improved image uniformity and better spatial resolution; however, they are more susceptible to motion artifacts [11]. The biggest challenges faced during the acquisition of high-quality diagnostic MRI sequences is the requirement of the infant or child to stay still for a more extended period. This is achieved by inducing sleep, providing friendly surroundings and other techniques. However, the majority of patients require sedation. Three-Tesla MRI is better at detecting and characterizing structural brain abnormalities in patients with focal epilepsy than 1.5 T MRI, leading to a better diagnosis and safer treatment of patients, according to a recent study. Three-Tesla MRI detected 65 of 74 cases, compared to 55 of 74 cases detected by 1.5 T MRI; lesions were accurately characterized in 63 of 74 cases using 3 T MRI, compared to 51 of 74 cases using 1.5 T MRI [16].

Insufficient clinical details from the referring neurologist has been touted to be one of the reasons for poor interpretation by the radiologist; also, studies have shown that the yield of MRI further increases by providing additional relevant clinical details to the radiologist, especially in the areas of suspicion; in addition, the experience and the expertise of the neuroradiologist play a vital role [17]. Standard evaluation by a neuroradiologist can detect differences of more than 20%; however, volumetric studies allow a more precise comparison and are particularly useful in the evaluation of patients with refractory epilepsy and negative MRI. Volumetric studies of the entorhinal cortex, for example, may identify occult damage ipsilateral to the seizure focus that is not evident on visual inspection [18]. The sensitivity of the inhomogeneous magnetization transfer technique for MS was highlighted by the reduction in the inhomogeneous magnetization transfer ratio in multiple sclerosis lesions and in the apparently normal white matter of patients compared with controls [19]. Magnetic Transfer Ratio (MTR) measurements not only provide semi quantitative information for Tuberous Sclerosis Complex (TSC) lesions but also reveal more extensive disease [20].

Other drawbacks of MRI in pediatric epilepsy are its high cost, lengthy scanning time, limited availability for various reasons and need of sedation. The field of magnetic resonance imaging continues to emerge with the use of a high magnetic field (7 T) and by combining it with other imaging modalities such as CT/PET scanners or better MRI/(F-labeled fluoro-2-deoxyglucose positron emission tomography)-PET (FDG-PET). By increasing the spatial resolution to microscopic values, the use of high-field MRI is promising for the diagnosis of Focal Cortical Dysplasia (FCD), and the use of 3 T in FCD has already reclassified almost 5% of cases which were previously diagnosed as cryptogenic with low-field strength magnets. The initial data obtained from the use of a high magnetic field 7 T in Gradient Echo (GRE) and Susceptibility Weighted Imaging (SWI) for the diagnosis of FCD are interesting and have potential to identify subtle cortical lesions [21]. De Ciantis et al., in his study of 21 patients with intractable focal epilepsy, demonstrated an improved diagnostic yield with the use of 7 T MRI for the detection of epileptogenic FCD which was not visible on conventional MRI [22]. However, the results need to be validated on a larger and more prospective sample before routine use. The concurrent recording of EEG/MRI also shows usefulness in the evaluation of intractable epilepsy [23].

### 4.1. Diffusion Tensor Imaging

Diffusion-tensor magnetic resonance (MR) imaging (DTI) and fiber tractography (FT) are the new techniques in the field of neuroimaging which demonstrate the orientation and integrity of white matter fibers [24,25]. DTI is an emerging MRI technique in the field of neuroimaging which uses anisotropic diffusion to show the integrity of the white matter axonal pathway. Fiber tractography utilizes the data collected by DTI and reconstructs the three-dimensional (3D) image of the neural tracts. DTI has been extensively studied for its application in white matter pathologies; however, it has a limited role in grey matter pathologies. Developmental central nervous system (CNS) diseases, both congenital and postnatal, can be a spotlighted field of DTI due to the potential for generating a fiber pathway and aberrant connections in the case of a blockage of normal white matter formation. It is recommended that DTT should be included in the routine procedures performed in the management of epilepsy. DTI allows the visualization of the exact location of tumors relevant to eloquent tracts and was found to be beneficial in neurosurgical planning and postoperative assessment; e.g., DTI allows the recognition of the optic radiation and helps to predict visual field deficits post-surgery [25,26].

### 4.2. Voxel Based Morphometry

Voxel-based morphometry (VBM) is a fully automated image-processing method which identifies differences in tissue density at a voxel level and also detects increases in gray matter concentration; however, it lacks pathologic specificity for FCD. Using different processing techniques for studying cortical thickness, the diagnostic yield for FCD has been significantly increased. With the use of computer-assisted systems, better images can be generated, which shows their potential for identifying lesions which were previously missed. However, the use of such systems and software need to be validated before they can used routinely [21].

## 5. Functional Neuroimaging Modalities in Pediatric Epilepsy

Functional neuroimaging modalities have progressed over the recent years in neuroscience and clinical neurological practice and play an important role in the diagnosis and treatment of epilepsy. Functional neuroimaging helps to localize the area of cerebral dysfunction, predominantly through changes in cerebral metabolism and the blood flow of specific brain structures and regions. While structural neuroimaging techniques such as MRI and CT scan identify underlying anatomical abnormalities, functional neuroimaging localizes the area of the abnormal cerebral perfusion and metabolic abnormalities and may potentially aid in the diagnosis and treatment of intractable epilepsy. Nuclear medicine imaging techniques such as SPECT and PET effectively identify changes in cerebral metabolism, perfusion and neurotransmission abnormalities during ictal, post-ictal and interictal periods. Functional neuroimaging is an important tool for localizing epileptogenic foci and helps in the presurgical evaluation of patients. About 33% of epilepsy patients do not respond to medical treatment; in these patients, the surgical resection of the epileptogenic foci becomes the only optimal treatment. Combining various neuroimaging techniques provides comprehensive information about the epileptogenic foci and surrounding functional area, which guides in the planning of potential invasive EEG monitoring and relevant surgery.

The functional neuroimaging modalities utilized in pediatric epilepsy include functional MRI (fMRI), single-photon emission computerized tomography (SPECT), positron emission tomography (PET) and magnetic resonance spectroscopy (MRS).

### 5.1. Functional MRI

Functional magnetic resonance (fMRI) imaging is a specialized MRI technique which demonstrates changes in blood oxygenation level dependent (BOLD) signals when the patient is engaged in an activity within the MRI scanner. fMRI is an evolving functional neuroimaging technique applied for epilepsy management and is mainly of importance for the pre-surgical evaluation of epilepsy patients who are candidates for surgery. The goal of such surgery is to remove only that area of the brain which causes seizures while preventing injury to the areas which control essential brain function. Functional mapping involves the localization of functionally critical regions, typically language, motor and the visual cortex [27,28], and their relation to the epileptic foci. For example, when the epileptic foci are close to the primary sensorimotor cortex, the precise localization of the activated region relative to the lesion can help to predict whether a sensorimotor deficit is likely to occur from foci resection [25]. fMRI can be used pre-operatively in localizing lesions relative to normal brain functions to decrease risks for postsurgical deficits and is increasingly applied for the functional mapping of the brain in patients who are candidates for surgery for intractable epilepsy. Currently, the primary use of fMRI in pediatric epilepsy is the mapping of eloquent function in candidates for resective surgery.

Through research and clinical experience, fMRI has been successfully used in the pediatric population and can even be applied in children as young as 5–7 years of age. The challenges faced while performing fMRI include the high level of anxiety, restlessness, and non-cooperativeness in the pediatric population [29]. Some studies suggest the use of noninvasive fMRI as an alternative to the invasive Wada test in the presurgical evaluation and in predicting post-surgical outcomes, and it is possible that in some institutions the Wada test may no longer be available [27,30]. However, the sensitivity and specificity of fMRI are not similar to the Wada test, especially in cases of extratemporal lobe epilepsy, hence requiring the Wada test [31,32]. Although fMRI can replace the Wada test in the majority of patients, the Wada test still needs to be done in patients with mental impairment and agitation for whom fMRI cannot be adequately performed [33].

Some studies have shown that the concurrent recording of EEG and fMRI provides additional details about ictal and inter-ictal epileptiform discharge, which helps in understanding the pathophysiology of epilepsy and identifies the location of the seizure onset zone. Simultaneous EEG-fMRI may achieve what seems otherwise to be largely impossible, namely the noninvasive recording of human brain activity with both high spatial and high temporal resolution [34]. The first applications of EEG-fMRI were born out of a clinical interest in the improved localization of the neural sources of epileptogenic EEG activity for diagnosis and presurgical planning [34].

### 5.2. Single-Photon Emission Computerized Tomography

Single-photon emission computerized tomography (SPECT) is a nuclear medicine imaging modality which uses gamma rays and allows the quantitative and qualitative assessment of regional cerebral blood flow [35,36]. SPECT provides imaging of the regional anatomy and monitors the level of biological activity (cerebral blood flow) at each place in the 3D region analyzed. The fact that there is increased ictal regional cerebral blood flow (observed by Sir Victor Horsley) or inter-ictal decreases in cerebral blood flow forms the basis for the use of SPECT in epilepsy. ^99m^Tc-HMPAO is a gamma-emitting tracer which is taken up in a manner similar to cerebral blood flow and has proven useful for functional neuroimaging in epilepsy. SPECT is not indicated in all patients with epilepsy but has an important role in the localization of epileptogenic foci and pre-surgical evaluation of patients, especially those with refractory epilepsy. Combining inter-ictal, ictal and post-ictal SPECT studies allows the accurate localization and lateralization of epileptogenic foci.

ISAS-ictal/interictal SPECT analysis by statistical parametric mapping (SPM) shows that ictal SPECT localizes the seizure focus and post-ictal SPECT lateralizes seizure focus (Chang et al., 2002; McNally et al., 2005) [37,38]. SPECT studies have proven value in temporal lobe epilepsy. However, its benefit is less understood for extra-temporal lobe epilepsy [39,40]. Subtraction ictal SPECT co-registered MRI (SISCOM) is a technique by which the interictal SPECT is subtracted from the ictal SPECT and the resulting image is co-registered with MRI. SISCOM has been shown to significantly increase the accuracy of SPECT [41]. SPECT is less costly compared to PET and is readily available [8]. The injection time is critical for ictal SPECT, and according to one study, an injection time of fewer than 20 s decreases the probability of false localization [42]. Additionally, Kudr et al. showed the importance of early radio tracer injection for successful seizure localization with ictal SPECT, and the study also mentions that factors such as the length of seizure, type of EEG findings and positive MRI may affect the extent of ictal SPECT hyperperfusion [43]. Krsek et al. analyzed a large population of pediatric patients with cortical malformations and showed that ictal SPECT is highly effective in localizing lesions of cortical malformation. The study also showed that SPECT predicted favorable postsurgical seizure outcomes following the resection of the localized cortical hyperperfusion. The study also suggested that the ictal SPECT can be a useful diagnostic tool in patients with epilepsy surgery failure. Although studies have shown that the complete resection of ictal SPECT hyperpefusion is associated with better post-surgical outcomes, ictal SPECT identifies a larger area of hyper perfusion compared to MRI and EEG-defined abnormities; hence, a larger resection may lead to a higher rate of complications. Other difficulties while performing octal SPECT in pediatric patients are that it may be difficult to recognize clinical onset of seizures, as well as the brief course and rapid spread of prevailing extra temporal seizure and the need for general anesthesia [44]. Of the 45 patients in whom a seizure focus was localized, a PET scan identified the same region in 25 cases (56% sensitivity) and SPECT in 39 cases (87% sensitivity) [45].

The traditional visual analysis of a SPECT involves the comparison of each cerebral region with the contralateral side. Limitations include difficulties in the detection of subtle changes, variations in the amount of injected radioisotope, the time of injection, and patient positioning. Also, the interpretation can be subjective, and if the epileptogenic zone is hypoperfused at the baseline (interictal), the ictal increase in tracer uptake may be obscured (i.e., it may appear normal) despite relative hyperperfusion [45].

### 5.3. Positron-Emission Tomography

Positron-emission tomography (PET) is a nuclear medicine imaging modality which detects gamma rays emitted by a positron-emitting radioactive tracer. The most common tracer used for neuroimaging is 2-deoxy-2 (^18^F) fluoro-d-glucose (FDG). This approximates the metabolic processes in the brain, providing a broad range of functional and metabolic information to help understand the mechanisms of neurologic diseases and guide therapeutic approaches. Most settings have used 2-deoxy-2 (^18^F) fluoro-d-glucose (FDG) in the interictal state [35]. A reliable ictal PET is often difficult to obtain as the half-life of the radiotracer used is extremely short. For patients with temporal lobe epilepsy, interictal studies show hypometabolism in epileptogenic areas. Interictal FDG-PET provides additional information about the epileptogenic foci to structural brain neuroimaging and can detect abnormalities in epilepsy patients, even those with a normal MRI. The sensitivity of interictal FDG-PET is high for temporal lobe epilepsy but is unclear for extratemporal lobe epilepsy. When a single region of metabolic abnormality corresponding to the EEG abnormality is detected on PET, such concordance helps to improve the surgical planning for controlling seizures and improving developmental outcome.

Ligand/neuroreceptor PET studies improve sensitivity and specificity for both temporal and extratemporal lobe epilepsy [46]. Neuroreceptor tracers show increased or decreased uptake in epileptogenic brain regions in the interictal state, unlike FDG-PET, which shows decreased uptake in the interictal period. Thus, this type of neuroimaging is of greater importance while detecting an epileptogenic area in the presence of multiple structural lesions, which confounds epileptogenic foci. ^11^C Alfa-methyl-l-tryptophan (11C-AMT) PET has been reported to effectively differentiate between epileptogenic and nonepileptogenic lesions in the interictal state in children with numerous-structural-lesion tuberous sclerosis. AMT-PET can also detect an epileptic cortex in non-tuberous sclerosis patients with normal MRI. ^11^C-flumazenil (FMZ)-PET involves benzodiazepine labeling [46]. ^11^C-flumazenil, the benzodiazepine receptor antagonist, has reportedly shown reduced binding in the epileptogenic focus. Flumazenil-labeled PET studies have been reported to be more specific than FDG studies, as the FMZ-PET image shows more restricted localization compared to FDG-PET. Muzik et al. showed a higher sensitivity of FMZ-PET compared to FDG-PET for the detection of cortical regions of seizure onset and frequent spiking in patients with extratemporal lobe epilepsy, whereas both FDG and FMZ-PET show low sensitivity in the detection of cortical areas of rapid seizure spread. The application of PET, in particular FMZ-PET, in guiding subdural electrode placement in refractory extratemporal lobe epilepsy will enhance the coverage of the epileptogenic zone [47]. Similarly, opiate, histamine, and *N*-methyl-d-aspartate receptor studies for epilepsy are under development [8,48].

### 5.4. Magnetic Resonance Spectroscopy

Magnetic resonance spectroscopy (MRS) is a technique by which a high-strength magnetic field is used which provides both structural and functional details of the brain. It gives a biochemical measurement of various brain metabolites noninvasively [36]. MRS demonstrates a reduction in N-acetyl aspartate (NAA) signals and increases in creatinine and choline signals. MRS is sensitive in showing biochemical abnormalities in specific epileptogenic foci in seizure patients for whom structural neuroimaging has failed to demonstrate lesions [36]. MRS has been used in the evaluation of both focal and generalized epilepsy. Magnetic resonance spectroscopy is indicated to screen pediatric epilepsy patients with metabolic derangements (inborn errors of metabolism) and for the characterization of masses detected on MRI. MRS yields additional details about the lesions detected on MRI, and it also identifies lesions which were missed with MRI. MRS has been shown to be of importance for patients with temporal lobe epilepsy and has prognostic significance for patients undergoing surgery for epilepsy; however, its sensitivity for neocortical and extratemporal lobe epilepsy is not well established [49]. Overall, MRS is used for the characterization of brain lesions but not for the localization of the epileptic foci or for improving the detection of subtle structural lesions.

## 6. Magnetoencephalography

Magnetoencephalography (MEG) is an electrophysiological technique which utilizes extremely sensitive magnetometers to record magnetic fields generated by the natural electric currents in the brain. This magnetic field can be used in mapping brain activity with the use of magnetometers. Magnetic source imaging (MSI) is a method by which the MEG is combined with other structural neuroimaging techniques such as MRI, significantly improving the yield in the management of epilepsy and providing a noninvasive option to study brain function and epilepsy [50]. MEG generates a high-quality temporal resolution within milliseconds, and in contrast to EEG, the magnetic fields are less distorted by the skull, cerebrospinal fluid, meninges and other tissues of conductivity [50]. Non-lesional neocortical epilepsies which have been challenging to identify could be diagnosed with MEG. It can localize epileptogenic foci which were not identified by structural MRI and assess the planning for intracranial EEG. MEG has high sensitivity and reliability for the localization of lesions and their relation to the functional cortex [35]. MEG can guide the placement of intracranial electrodes in cases which requires invasive EEG.

Cortical dysplasia and mesial temporal sclerosis, which are important causes of refractory epilepsy in pediatric patients, are often undetected by conventional imaging modalities; MEG can provide useful information about these lesions and help in their management. It has proved to be an important tool in epilepsy management, helping to gather essential information pre-operatively. MEG has higher sensitivity for dipoles tangential to the surface, while EEG has higher sensitivity for radial dipoles. Thus, an abnormality undetected on EEG can be detected by MEG, and vice-versa, and so simultaneous EEG and MEG recordings can have superior sensitivity in comparison to studies done alone. This is of particular importance for neocortical epilepsy [35,51].

## 7. Multimodal Approach

Numerous techniques are available for detecting the structural and functional abnormalities of an epileptogenic focus. One of the breakthroughs in the field of neuroimaging for managing epilepsy is the simultaneous integration and co-registration of various imaging techniques. The multimodal approach involves the integration of various imaging modalities such as MR imaging, diffusion tensor imaging, MR/FDG-PET fusion imaging, SPECT and magnetic source imaging [52,53]. These neuroimaging modalities may each have strengths and weaknesses and measure different aspects of brain structures and functions in the ictal and interictal states; thus, they cannot replace each other, but rather are complementary to each other. However, the combination of modalities used to effectively localize the epileptic foci needs individualized optimization on a case-by-case basis.

The diagnosis and treatment of intractable epilepsy has been of significant interest in the pediatric population, and advances in such neuroimaging techniques and the implementation of a multimodal approach have dramatically improved potential clinical applications, including guiding surgical treatment. Multimodal neuroimaging approaches play an essential role in the non-invasive localization of epileptogenic foci and have an established role in guiding the management of epilepsy [53].

PET/MRI hybrid imaging has evolved as an exemplary multi-modal approach for the evaluation of epilepsy. Several studies have shown improved diagnostic yield for potential epileptic lesions using hybrid PET/MRI [54,55]. The co-registration of FDG-PET/MRI helps in localizing seizure foci noninvasively and in the successful surgical treatment of patients with cortical dysplasia (CD), especially for patients with no concordant findings and an apparently normal MRI scan [52,56]. Chassoux et al. demonstrated the high sensitivity of FDG-PET co-registered with MRI in detecting intractable partial epilepsy syndromes such as Taylor-type focal cortical dysplasia (TTFCD), greatly improving the diagnosis and surgical prognosis of patients with negative MRI [57]. Similarly, Desarnaud et al. showed that the integration of electroclinical data and FDG-PET/MRI co-registration have a high localizing value in type-2 FCD, and this approach may also help in improving surgical outcomes in extra temporal epilepsy, even in patients with negative MRI [58]. Studies have shown that the diagnostic accuracy of PET/MRI for seizure focus was not inferior to PET/CT [59]. In an interesting study by Grouiller et al., in which they utilized a single session quadrimodal approach for the pre-surgical interictal imaging of pharmacoresistant focal epilepsy, combining FDG-PET, MRI, simultaneous EEG-fMRI and EEG-based electric source imaging (ESI) in a single session using hybrid PET/MR scanner, this multimodal approach was found to result in improved workflow and efficiency while reducing costs and time demands without compromising the diagnostic yield [60].

## 8. Conclusions

Recent advances in neuroimaging have revolutionized the management of epilepsy, especially in refractory epilepsy. Structural neuroimaging continues to evolve with advances in MRI techniques, making it possible to scan previously undiagnosed lesions. CT scans still have an important role for diagnosis in acute emergency conditions and in cases in which MRI is not available. Recent advances in MRI scanning, including DTI and tractography, allow the visualization of white matter tracts. Functional neuroimaging can provide detailed information about the epileptic focus. While invasive intracranial EEG monitoring is considered to be the gold standard for the localization of epileptogenic foci and functional mapping in resective surgery, there are many potential complications; neuroimaging techniques such as fMRI, PET, SPECT, MEG, and MRS have limited the use of invasive intracranial EEG without worsening surgical outcomes, and in cases in which intracranial EEG is performed, they assist in planning the placement of intracranial electrodes. Functional MRI allows the non-invasive functional mapping of various brain regions, decreasing the risk of functional deficits. Functional neuroimaging modalities such as fMRI, SPECT and PET have a definite diagnostic role in the presurgical evaluation of patients, helping in the pre-surgical evaluation and assisting in predicting surgical outcomes. A multidisciplinary approach combining such anatomical and functional neuroimaging studies has significantly improved the ability to develop management strategies for intractable epilepsy in the pediatric population. However, a significant knowledge gap still exists in the management of pediatric epilepsy, including both temporal and extra-temporal lobe epilepsy. Also, there is a need for universal guidelines for using the above-mentioned neuroimaging techniques.

## Figures and Tables

**Table 1:** Indications for structural neuroimaging in pediatric epilepsy.

Indications
History, physical examination, electroencephalograph (EEG) changes, and clinical evaluation suggesting localization-related epilepsy. Generalized epilepsy syndromes that are known to potentially have focal lesions, as seen in Lennox Gastaut syndrome and infantile spasms. Abnormalities on neurological examination including focal neurological deficits, neurocutaneous stigmata (Sturge–Weber syndrome, neurofibromatosis, tuberous sclerosis, epidermal nevus syndromes, etc.) and dysmorphic conditions such as microcephaly or macrocephaly. Cerebral malformation syndromes (focal cortical dysplasia, hemimegalencephaly, gangliogliomas and dysembryoplastic neuroepithelial tumors (DNET), lissencephaly, etc.) Seizures changing characteristics, causing developmental regression, uncontrolled or worsening seizures. History or physical examination suggestive of substantial developmental delay, arrest, or regression. New onset seizures with signs of medical emergencies such as increased intracranial pressure or status epilepticus.

**Table 2:** Advantages and disadvantages of MRI vs. CT.

Magnetic Resonance Imaging	Computed Tomography
**Pros:** Imaging modality of choice. High sensitivity. Low radiation risk. Detects small/subtle cortical abnormalities and temporal lobe abnormalities (e.g., mesial temporal sclerosis)	**Pros:** Used for assessment in emergency conditions. Fast scanning speed. Accessibility. Easy to use. Comparatively lower cost. Sedation not required. Better than MRI for calcified lesions (congenital infections) and neurocutaneous malformations (e.g., Sturge–Weber syndrome, tuberous sclerosis)
**Cons:** Sedation required. Slow scanning speed. Limitations in accessibility (less-developed countries). Comparatively higher cost. MRI cannot be done in presence of dentures, pacemakers and other metallic implants. Motion artifacts (esp. with 3 T & 7 T)	**Cons:** Risk of radiation exposure. Low-resolution images. Low sensitivity (30%). Limitations in detecting some pathologies of temporal fossa such as mesial temporal sclerosis and small/subtle changes

**Table 3:** Recommended MRI epilepsy protocols.

Protocols
Standard thin slice T1-weighted gradient-recalled-echo sequence. Axial and coronal T2-weighted fast spin-echo or turbo spin-echo sequences. Axial and coronal Fluid Attenuation Inversion Recovery (FLAIR) sequences. Three-dimensional (3D) T1-weighted volume acquisition sequences. Oblique coronal T2-weighted imaging of the hippocampus. For children younger than two years of age: 1–2 years of age: Axial, coronal and sagittal T1 weighted sequences. <1 year of age: High-resolution Axial, coronal and sagittal T2 weighted sequences.
